# Israel's civil society 2023 from protest to aid provision – a serving elite perspective

**DOI:** 10.3389/fsoc.2024.1417687

**Published:** 2024-07-09

**Authors:** Benjamin Gidron, Hagai Katz

**Affiliations:** Guilford Glazer Faculty of Business and Management, Ben-Gurion University, Beersheba, Israel

**Keywords:** serving elite, democracy, protest, emergency services, Kibbutz, National-Religious movement, liberalism

## Abstract

The year 2023 in Israel started with illiberal constitutional change efforts by the new right-wing government and concluded with a brutal attack by Hamas terrorists and the subsequent war. Both occurrences galvanized two massive surges of civil society activism. The first was a mass protest that impeded the government's undemocratic legislation. The second was a large-scale mobilization to support a variety of populations affected by the war, providing services and goods that supplanted the failed governmental crisis response. Using a *Serving Elite* perspective and elaborating on this concept in the Israeli context, the paper analyzes the organizations that transitioned overnight from protest to service delivery. While these are two known roles played by civil society in general, such a transition from protest to support within the same organization is unusual, if at all existent. The paper analyses nineteen (19) in-depth interviews with leaders of 10 prominent organizations active in the protest and the aid phases. It explores their participants, forms of organizing, activities, ideologies, and interconnections, particularly emphasizing the transition. Thematic analysis of the interviews revealed the emergence of a new *Serving Elite* in the making, liberal in orientation, and developed during the crises. This perspective provides an opportunity to highlight processes deeply embedded in Israel's social, political, and cultural landscapes, changing elites and power relations, and Israel's culture of entrepreneurship. It also provides a framework for analyses of serving elites in other countries.

## 1 Introduction

The year 2023 was particularly dramatic in Israel. It started with illiberal constitutional change efforts by the new right-wing government and concluded with a brutal attack by Hamas terrorists and the subsequent war in Gaza. Both occurrences galvanized massive surges of civil society activism. The first was an unprecedented mass protest that impeded the government's undemocratic legislation. The second was a large-scale aid response providing services and goods to various populations that supplanted the failed governmental disaster response.

The paper focuses on the active organizations involved in the protest, most of which transitioned overnight from protest to disaster aid. While protest and service provision are two known roles played by civil society in general, and sometimes a service provision organization takes on the role of advocacy, a transition from protest to aid *within the same organization* is practically unheard of. The need to rapidly change organizational orientation highlights the dynamic nature of its management and strategy.

We chose to analyze this phenomenon from a *Serving Elite* perspective, which focuses on the participants' self-perception as an elite, with critical responsibilities to preserve both the (democratic) nature and the security of the society and the country. The paper first analyzes the concept of *Serving Elite* and reviews its development and changes in the Israeli context since the early 20th century. It then briefly describes the dramatic events during 2023, especially the transition from protests against the proposed “judicial reform” (which constituted a regime change) to the war in Gaza. It also introduces the major organizations that were active in both events. Its Findings section analyzes in-depth open interviews with 18 leaders of 9 organizations. We asked them to talk about themselves (personal and professional background), the organizations they led and their activities, and additional thoughts about the pre-war protest and the Gaza war. An analysis of these interviews reveals six themes for our interviewees, the major undercurrents of their activities in both the protest and the support efforts. Lastly, we discuss these themes from a *Serving Elite* perspective and suggest that the events of 2023 gave birth to a new, Liberal serving elite in the making. Based on our findings, we propose an empirically-based conceptualization of the concept that can assist in studying this phenomenon and comparing it across time and place.

## 2 Literature review

### 2.1 Serving elites

A recent article in an Israeli newspaper dealing with elites opens with the following statement:

The concept of “elite” or “elites” is much vilified these days. One can safely assume that it has never been popular with ordinary people who disliked the notion that, to use the Cambridge English Dictionary's definition, there is a group of people that is “the richest, most powerful and best educated, or best trained in a society.” (Jacob, 2023)

Indeed, this is how elites are perceived not only by journalists but by researchers as well. Khan (2012) defines elites as “actors who have vastly disproportionate control over or access to a resource that is valued by others” (p. 362). While in a society where everyone is equal, the idea that some persons have more privileges than others is unacceptable, the other side of the coin is that certain people must have the power to make decisions for society at large (or segments thereof) and from that position they can be viewed as “elites”. We are not talking about elites by birth, such as nobility or members of a caste system, who inherited privileges based on their family roots, but about groups of people who enjoy a variety of physical, cultural, and symbolic resources, which in turn give them more prestige and power than other members of society (Ben Rafael and Sternberg, 2007). Such elites can set norms and behavior patterns and influence policies. Accordingly, existing elites sometimes develop *counter-elites* based on competition over material resources or ideological conflicts. The issue of values is critical in the discourse of elites as these suggest that agreement between the values of elites and the rest of the population is essential for the smooth management of society (Ben Rafael and Sternberg, 2007).

The study of elites and elitism was a major topic of inquiry by sociologists and political scientists during the first half of the 20th century. Questions such as who the decision makers are, who shapes the reality in society, how this elite acquired its position, and what are its relationships with the people they influence—have been the focus of classical works by Lasswell (1934), Pareto (1935), Mosca (1939), and Michels (1962), to name a few. The more recent literature on the subject widens the scope and inquires how elites access a social resource (Khan, 2012): “Control over or access to transferable social resources provides elites with disproportionate social power and advantages; their decisions and actions influence and affect vast numbers of people”. Khan does not talk about one elite but several in different domains, categorizing them by their resources and the decisions they control in the different arenas: Political, cultural, economic, academic, religious, etc. He adds that after the rights movements of the late 1960s, interest in elites waned…[yet] more recently, work on elites has become popular again.

Korsnes et al. (2017) demonstrate this renewed interest in studying elites by examining various forms of capital that elites mobilize, such as economic, cultural, and social. In their book, they provide international comparisons of those. Other examples are studies that focus on the British Senior Civil Service (SCS) elites and that many of SCS's employees have graduated from private schools, emphasizing the “Oxbridge” route (Dowding, 1995; Talbot, 2014; Elitist Britain, 2019). This course emerged during the colonial era but managed to sustain itself. Such a perspective of elites in the service of the public is shared by Goldreich (2014), for whom the term “elite” relates to those who staff key positions in public institutions, including judges, academics, etc. What guides these individuals is the public interest that serves the entire population as they understand it. He adds that when private interests lure the public-serving elite to abandon their fundamental values and replace them with private benefits—society has a significant problem. In that sense, elites do not just have privileges; they primarily have responsibilities.

This emphasis on the public interest and those who protect and promote it is encapsulated in the “serving elite” concept. Unlike traditional views of elites centered on privileges, the serving elite concept prioritizes societal responsibilities. In this sense, Israel is a good example: the notion of a select group of individuals devoted to national service in government, the military, business, or the religious establishment has been firmly rooted since Israel's pre-state era. It manifested itself in certain processes at particular timeframes, giving rise to specific serving elites who saw themselves in societal leadership positions. They preached specific directions for society's preservation and development. They also acted, often taking real risks and even willing to sacrifice their lives, setting an example and paving the way for others to follow.

Early mentions of the concept in Israel appear in discussions of the role of education (Shapira, 1984) and youth movements (Gouri, 1989), deemed as the agents whose role is to develop an elite that is not self-serving but rather acts in the service of the public good. The Socialist-Zionist thinker and educator Ben Aharon (2012) identified the serving elite as a tenet of the Jewish pioneers in Israel's pre-state era and pointed out the Kibbutz movement as the apex of this notion. The discussions of the religious Zionists as a serving elite find their justifications in Jewish thought, as in the service roles of the Temple priest (Ben Admon, 2013). A parallel to this concept of religious service and leadership is the idea of Samaritan Leadership, which is based on the ideal of service that is common to many religions and civilizations (Egan, 1994; Folkman, 2018). The servant elite concept is closely related conceptually to servant leadership (Greenleaf, 2002), who defined it as an extreme prototype of a leader who wants to serve first and then consciously chooses to assume a leadership position to achieve that. Thus, leading is a form of and a venue for service. Addressing graduates of the Shalem College^1^ in June 2017, Haim Gouri, a leading poet, had the following definition for the concept “elite”: “An ‘elite' is a group of individuals that contributes in different ways to culture, to national resilience, and to universal values, and lives a deeply moral existence at the heart of its society” (Jacob, 2023).

### 2.2 Israel's serving elites

An analysis of Israel's history from a serving elite perspective will disclose two clear examples of this phenomenon rooted in different ideological contexts: (1) The Kibbutz movement—a collective egalitarian community with roots in the Socialist ideology of the late 19th century, which found expression, starting in 1909 (Kibbutz Degania), in hundreds of Kibbutzim in many parts of Israel. In the 1980s, against the background of an economic crisis as well as processes of individualization, it saw a decline in its role as a beacon for society and of its members as a serving elite. (2) The National-Religious movement—developed after the 1967 war and received an extra boost after the 1973 war. It finds its roots in the teaching of Rabbi Kook, who saw the victories of these wars as signs of redemption and the upcoming Messianic age, which calls for settling the entire Land of Israel to hasten the coming of the Messiah. Similarly to the Kibbutz movement, whose members settled the land in outposts, far from urban centers, the National-Religious movement has been building settlements outside of Israel's official and recognized borders—in the occupied territories. We claim that the dramatic events in Israel during 2023—the attempted regime change and the war in Gaza that mobilized civil society, first to protest and later to support different affected populations—are the potential birthplace of a third serving elite—a Liberal one.

#### 2.2.1 The Kibbutz movement as a serving elite

The Kibbutz concept and its implementation have been the subject of thousands of studies and publications from various academic disciplines. Based on national and social ideals, it attempted to create an exemplary community that served the individual and the nation. Achouch (2023) depicts the kibbutz's qualities: “It has been simultaneously a revolutionary movement, a network of rural communities, a town planning alternative distinct from city and village, and an avant-garde of Zionist nation-building” (p. 1). Members of the Kibbutzim were perceived as pioneers, leaving a comfortable city life and risking their lives by choosing to live in an outpost, often near the border, engaging in physical labor, and creating an egalitarian, democratic community. Shapira (2010), a leading Israeli historian, discusses the national role of the movement:

During the period of the British Mandate, the kibbutz had always been the standard-bearer, ready to establish “tower and stockade” footholds in dangerously exposed territory or to volunteer for whatever else needed to be done… After the attainment of independence in 1948… new kibbutzim were still being set up along the borders... Kibbutz members, more than other citizens, volunteered for elite units, flight squads, and the commandos—a price tag of blood held up by members or outside supporters in the face of any criticism flung at the kibbutz.

Near (2011) and Drory (2014) write about the disproportional contribution of Kibbutz members to Israel's security, for which society rewarded them. These rewards included close ties with the Labor Party-led governments (1948–1977) and representation in various political posts during these years, far exceeding their proportion in society. According to Near:

In the changed circumstances of the State of Israel, the concern for security was (and still is) epitomized by the part played by kibbutz members, and especially the younger generation, in the Israeli Defense Forces. In both cases, the kibbutz has contributed to the physical security of the wider community far beyond its numerical proportions; and it is rewarded in a variety of ways, from prestige to political and economic support… For the kibbutz has always prided itself on its contribution to Israeli society, and considered itself to have earned whatever special status and privileges it has by this contribution... The kibbutz, in this view, is, and aims to be, a *serving elite*. (emphasis added, p. 210)

In the 1980s, the idealized image of Kibbutzim as a societal role model with values of equality, communal decision-making, and hard labor no longer fit the reality in which Israel found itself. Facing a right-wing government, often antagonistic to the Kibbutz movement and economic pressures, the Kibbutz movement shifted from agriculture to industry and later to high-tech, abandoning its original ideals, focusing now on the individual and practically ending its role as a serving elite. Shapira summarizes the demise of the original Kibbutz ideals:

Ironically enough, it was the economic collapse of considerable parts of the kibbutz movement in the late 1980s that disrupted this drab state of affairs. The principles of egalitarianism and communalism were sacrificed on the altar of economic efficiency. The collapse of the USSR resonated powerfully: economics had defeated ideology, and the expert in social organization replaced the charismatic leader. The aspiration to reshape the world was replaced by a far more modest concern: to ensure a dignified old age, at least for the generation that had given its all for the kibbutz. (Shapira, 2010)

Achouch depicted that same process (2023, p. 1–2):

[From the 1980s] Kibbutzim appeared among the affluent elite class in Israeli society. In the process, the kibbutz's utopian goals… had come up against the political contingencies and contradictory evolutions of Israeli society. After being a model of humanist socialism for the entire Western world during the 1960s and 1970s, the kibbutz movement suffered an unprecedented socioeconomic crisis during the 1980s and 1990s.

With the decline of the Kibbutz movement and against the background of Israel's undefined final national borders and unsettled security problems, a new National-Religious serving elite developed.

#### 2.2.2 The national-religious movement as a serving elite

The Israeli religious community in Israel is divided into two major camps: (1) The Ultra-Orthodox, which does not recognize non-religious Jewish thinking and life„ focuses on fulfilling the personal and communal Torah commandments only, lives in their own neighborhoods and wait for redemption with the coming of the Messiah; and (2) the Religious National Movement, which has its feet in both the world of the Torah and the secular culture. Its members participate in politics and business; they serve in the army and are otherwise involved in society. Central to the movement is its relation to the Land of Israel (Eretz Israel). Accordingly, this is not only the homeland of the Jewish people but a holy site, which entails the commandment to settle the land. Rabbi Kook (1865–1935) founded the movement. In the early part of the 20th century, he refrained from criticizing the secular Kibbutzim and saw them as a first sign of redemption: The work of those who settled the Holy Land is holy:

*Eretz Yisrael* [the Land of Israel], Kook wrote, was the spatial center of holiness in the world, radiating holiness vertically to the Jews who lived upon the land as well as horizontally to other Jews and peoples of the earth. The spirit of the land was entirely pure and clean. The air of the land really did “make one wise,” as the Rabbis had said (Eisen, 1986).

The six-day war (1967) served as proof that affirmed for the Religious National Movement its ideology. They saw Israeli victory in that war and the return to the Holy Sites of Jerusalem, Hebron, and Bethlehem as a miracle and a sign that redemption was on its way. Building on this ideology, after the 1973 war, and with the encouragement of Rabbi Kook's son, a settlement movement in the Occupied West Bank (called by the settlers Judea and Samaria) started. In a parallel move, the movement established Torah educational institutions, mainly at a high school level and often with boarding facilities, to nurture the next generation of National-Religious supporters. In addition, they established research institutes and philanthropic funding sources to bolster the National-Religious ideology (Sharlo, 2007).

Like the Kibbutz example, the National-Religious Movement considers its members as pioneers and a serving elite for the people of Israel. This manifests clearly in the fact that their members volunteer to serve in the fighting units in the army and their activity as settlers in the West Bank. These two activities represent a commitment that involves danger and risk. In addition, the National-Religious Movement is attempting to “educate” the non-religious population and has developed Torah-oriented Nuclei (Gar'inim Toraniyim). These are groups of members of the movement, usually young couples, who rent several apartments in low socioeconomic neighborhoods or towns, attempt to be involved in the local community by engaging in volunteer work, and indirectly try to “proselytize” the population to become more religiously observant and nationalist (Cohen and Billig, 2023).

This multi-faceted involvement of the National-Religious Movement in various aspects of public life is made possible by their political power, as expressed by their representation in the Knesset and particularly in the right-wing governments, which started in the late 1970s. Whereas, the Kibbutz Movement in the Pre-State era and into the 1970s gave birth to a serving elite that provided an ideal based on social democracy, the National-Religious Movement gave birth to a serving elite that has as an ideal the settlement in the entire land as a process of hastening the coming of the Messiah.

#### 2.2.3 The events of 2023 and the evolution of a potential liberal serving elite

In November 2022, Israelis went to the polls for the 5th time in 3 years. The result was a coalition government composed of the Likud and three religious parties, forming the most right-wing government in Israel's history. That government had a small majority, so it depended on its religious partners.

One of the first items on the new government's agenda was a “Judicial Reform” led by the Minister of Justice. He planned to pass several bills and take other administrative measures that would annul the Supreme Court of its power to criticize the government based on the reasonability of its decisions. Additionally, he proposed changing the system that appoints judges and gives the coalition a majority in the appointing committee. Parallel to these proposals, there were attempts by politicians to control the processes of appointments of senior public administrators and provide them with the power to appoint their senior staff, thus undermining professional principles in public service administration. The result of all these proposals would have shaken to its foundations the fragile separation of power between the executive, legislative, and judicial branches and create a de facto authoritarian state, similar to Hungary or Turkey, where all the power of the state rests with the executive branch.

On the week the Minister of Justice announced his plans, a large demonstration was spontaneously organized in Tel Aviv to oppose it. In the following weeks and months, demonstrations were held regularly with hundreds of thousands of participants, usually on Saturday evenings in all parts of the country. The target was the “Judicial Reform,” which the protesters termed “Regime Change,” and the demand was to scrap it. In addition, other measures were taken by those opposing the government's plans, such as pleas to the Supreme Court, continuous harassment of coalition members near their residences, a blockade of the Ben-Gurion International Airport, and more, involving increasing numbers of protesters.

Over time, two categories of participants emerged: While many protesters came as unaffiliated individuals, most came based on belonging to specific groups and organizations, wearing their shirts, and assembling together in the demonstrations. The most significant ones were *Brothers in Arms*—consisting of veterans from the Army, Navy, and Air Force as well as from other security agencies; these, in turn, were subdivided into specific units—artillery, infantry, etc., which consisted of “buddy systems”—friends with long term close relationships; *Building an Alternative (Bonot Alternativa)*—a feminist organization, *No Academy without Democracy*—academics, *No Education without Democracy*—teachers and parents of schoolchildren; *The White Coats*—doctors and health professionals, *The Black Gowns*—lawyers, *The Hi-Tech Protest*—entrepreneurs and personnel in startups, *LGBT for Human Rights*, and more. Each group protested based on the danger of the proposed judicial measures to its professional and personal safety in an undemocratic context. While many of these organizations did not exist before and arose in response to the “Reform,” they turned out to be well-organized, based on WhatsApp communication,^2^ and could call on members to attend activities on the spur of the moment. They stressed the fact that they do not use violence. Indeed, clashes with the police were minimal throughout the protest (January–September 2023). An additional layer of protesters was the local or regional one. While the focus was the big demonstration in Tel Aviv on Saturdays, there were some 50 local demonstration foci around the country, with local persons responsible for their operation (choosing the speakers, building a stage, putting loudspeakers, etc.). This operation had an overall Coordinating Committee—consisting of former politicians, funders, and other public figures. They were mostly engaged in fund-raising for the protest efforts and usually kept out of the limelight. However, clarifying that they didn't directly manage the events is essential. Instead, individuals drove the initiatives, facilitated by a communication network within WhatsApp groups.

On Saturday, October 7th, at the end of the Jewish High Holiday Season, the plan was to have another series of big demonstrations nationwide. The events of that morning changed those plans. In a dramatic turn of events, the protesting groups, led by *Brothers in Arms*, changed their orientation overnight and opened headquarters in a big shopping center on Sunday morning. Within days, the headquarters divided into specialized branches and began collecting and providing a growing range of services. These initiatives evolved gradually in response to expressed needs: One branch collected personal equipment for the reserve soldiers that were drafted and clothing for the evacuees from the towns and Kibbutzim around the Gaza Strip and from the North; another managed a system of transportation for individuals who needed evacuation and organized residences for them; yet another undertook identifying kidnapped or missing individuals from different sources of intelligence; another mobilized volunteers to help farmers who lost their (foreign) workers that left the country, to name a few of those initiatives.

This immediate change of orientation of civil society organizations focused on protest into those who provide services to various populations in different locations is remarkable. It may not be surprising that during times of emergency, the public sector is slow to react, and civil society often takes its place, at least in the initial phases (Eikenberry et al., 2007). In Israel after October 7th, the government not only failed to protect the lives of its citizens around Gaza but also failed to provide immediate support for those affected by the Hamas attack. Notably, other civil society organizations not part of the protest were also involved in the efforts to serve those in need because of the war. There were also some organizations involved in the protest that refused to work to support the affected population, claiming this was the duty of the government. Still, what we find significant is the change in orientation of those who moved from protest to support, which is our paper's focus.

Looking at these events and processes from a *serving elite* perspective, undoubtedly, in 2023, Israel experienced two very significant crises, which called upon a massive mobilization of civil society. Initially, it was an urgent call to protect the democratic nature of society. Later, it was a concerted effort to address the pressing needs of various affected populations when the public sector proved incapable and slow to react. Given the centrality of civil society, which “performs key functions in liberal democracies” (Johansson and Meeuwisse, 2024, p. 2), we argue that the groups that protested for 9 months in defense of democracy and later moved to protect different populations affected by the war, represent a new serving elite—a liberal one—that has a significant role in shaping the nature of the post-Gaza War Israel. An analysis of the groups that participated in these two processes shows that they consist of people who lead the economy and security of the country, as well as the professions. They are indispensable for the survival of a democratic, progressive society, part of the Liberal Western world. These civil society groups are, in effect, a *Liberal serving elite in the making*. The values they promote—human rights, equality for all, personal freedoms, protection of democratic institutions, and finding a solution to the Israeli-Palestinian conflict—the values that called them to action in 2023 are the base of that would-be elite.

We can see this new *Liberal serving elite in the making* also as a counter-elite to the *National-Religious serving elite*. Activities of the former on the two fronts—protest and aid, reflect its contestation (Johansson and Meeuwisse, 2024) of the values promoted by the latter, which the 2023 government backed. In that sense, the ideological conflict between the two serving elites reflects the political chasm in the Israeli population.

## 3 Methodology

The study used a qualitative research method based primarily on open interviews. We interviewed 19 leaders of 10 organizations during January 2024, with 1–2 interviewees per organization. In addition, before and after the interviews, we read written material by and about the organizations we covered. The interviews were carried out via videoconference when the interviewee was at home or in the workplace. The rationale for using videoconference rather than face-to-face interviews had to do with the fact that they were conducted during an emergency time (the war) with possibilities of interruptions by rocket fire resulting in limitations of transport. While these rocket attacks did not take place on a daily basis, the idea was to have a unified system for all interviewees. The duration of the interview typically ranged from an hour to an hour and a half. Researcher 1 led the interview, while Researcher 2 asked supplementary questions and took field notes. The interview began by introducing the researchers and the study's background and purpose. Then, consent was obtained to participate in the study and to record the interview. We recorded the interviews using Zoom's built-in recording system. In some cases, when available, we used the interviewee's previous recorded interviews in the media.

The interview protocol consisted of three open questions:

1. Opening question: Please tell us about your organization from the beginning until October 6th (the protest phase).
2. Follow-up question: What happened since 10/7 (the start of the Gaza war)?
3. Follow-up question 2 (as needed): What are the plans for the organization from now on?

We let the interviewees speak freely, and when necessary, we asked them to clarify or expand their answers.

We transcribed the interviews using Hebrew-enabled AI (thetranscriber.net) with human review. We analyzed the texts thematically following Braun and Clarke's (2006) process for a structured thematic analysis while maintaining the flexibility inherent to this method. We analyzed the texts at the explicit (or semantic) level (Boyatzis, 1998), also called naïve-realist (Madill et al., 2000). We applied repeated reading (Braun and Clarke, 2006) to identify key and repeated arguments within and between the interviews and grouped them into themes. We each coded separately and then compared and combined our analyses into one code system. The analysis was assisted by Quirkos©, version 2.5.3 (Turner et al., 2024). We conducted and analyzed the interviews in Hebrew; the quotes below are our translations of the original statements.

Besides the interviews, the study also used four more sources of information:

1. Field notes from researchers' participant observations in multiple protest events, demonstrations, and support activities of a few target organizations.
2. Observations in the headquarters of one of the larger organizations.
3. Written material of and about the organizations, including news interviews, blog posts, and websites.
4. Previous participants' interviews that were recorded in the media.

For a list of organizations whose leaders^3^ we interviewed, see Table 1. Detailed descriptions of the organizations and their activities are in the Appendix.

**Table 1:** Organizations studied.

**Brothers and Sisters in Arms (Achim La'Neshek)**	**Organization of reservists from the IDF, as well as other security forces**.
Women Building an Alternative (Bonot Alternativa)	A feminist activist organization that seeks to promote gender equality, empower women, and raise awareness of violence against women.
No Academy without Democracy	Academic leaders from all universities, colleges, and other scientific institutions
No Education without Democracy	Organization of educators, parents, and students who demonstrated outside educational institutes.
White Coats Health Professionals	An organization of doctors, nurses, and other health professionals led by the chair of the doctors' union.
High Tech Protest	Leaders of the Hi-Tech community concerned about the consequences of the “reform” to the future of that industry.
Local Centers of Protest (two centers)	In over 60 locations outside the main cities, regional nuclei organized protests in parallel with the main cities, with similar content.
Crime Minister	Protest against Prime Minister (PM) Netanyahu, focusing on the fact that the prime minister is on trial and accused of corruption.
Black Flags/Kaplan Force	A protest organization against PM Netanyahu focused on his corruption, later renamed Kaplan Force.

## 4 Findings: thematic analysis

In analyzing our interviewees' responses, we identified six major themes that, according to our respondents, were the forces that pushed them and their organizations and characterized their activities. These can be divided into three dimensions, as detailed in Table 2. We present the quotes by the interviewees' organizations instead of their names, as requested by them.

**Table 2:** Major themes.

**Dimension**	**Theme**	**Description**
Personal traits	(1) Personal and professional identities	Encompasses the activists' personal and professional identities, which may be threatened in authoritarian regimes and must be protected
	(2) Ability to accept plurality of ideas	Refers to the activists' recognition of the deep societal divisions in Israel and the need to foster a cohesive Israeli identity
Belief systems underlying activity	(3) Guiding values and ideologies	Includes the values and ideologies guiding the respondents, such as support for democratic regimes, human rights, equality, and rejection of authoritarian systems
	(4) Attitude toward the occupation	Reflects the activists' attitudes toward the occupation and their stance regarding authoritarian regimes
Commitment to society and country	(5) Leadership—preparedness to contribute to the public in a variety of ways	Highlights the activists' readiness to serve the public through various means
	(6) Patriotism	Indicates the activists' dedication to their country and society, evident in their active involvement in both protest and war efforts

### 4.1 Personal traits

#### 4.1.1 Professionalism/personal identity

In the academic headquarters, we are closely connected to the doctors, the mental health professionals, and ‘the Black gowns' (lawyers' organization) - the people who organized based on professionalism. It was a good way to make friends. I also now work a lot with high-tech activists, but it's under my academic hat. *No Academy without Democracy*

Democracy experts explain how the proposed ‘judicial reform' could have significantly weakened democracy. A strong democracy is essential to national security, similar to the high-tech people who declared that a strong democracy is vital for high-tech development and investments.

If someone thinks that he will put his military boot on our neck and tell us, “I am the majority, and we decide,” without any checks and balances, he will suddenly discover that the real majority - most of the people who contribute, including reservists, have no choice but to stand against such moves. *Brothers in Arms*

A major common denominator for membership in the groups active in the protest was the professional identity that defined them and justified their protest. Indeed, a non-democratic regime suppresses creativity and freedom of expression, which will likely harm professional thinking and identity.

A lot of men who consider themselves liberals don't put women around the decision-making tables. Sometimes, it's easier to identify the chauvinist men on the right. The men on the left are more sophisticated. They pretend to be enlightened, but in fact, they do not meet the full definitions of liberalism. So, as a women's organization, gender equality is another arena of our struggle that other protest organizations do not have to deal with.

Our overarching goal is political, feminine, liberal, and activist power. The secondary goals are female representation and financial and personal security. It hasn't changed; these things are guiding us. *Building an Alternative*

This is also true of personal identity. In a society guided by religious principles, women have an inferior position, which needs to be countered by social and political action. The quote regarding other women's organizations not joining the protest targets organizations that provide services to women (and are supported by the government) but do not engage in political action to protect their status as women.

I'm optimistic because, as far as I'm concerned, we've already won. The road there can be terrible with crashes or problems, but this block is going to dictate the tone; it's actually the block that supports this country; it's the block that serves it. The ‘legal reform,' the ‘regime change,' whatever you want to call it, caught me in a place where I realized, not immediately, but within a few weeks, that if this legislation passed, I would not be able to live my life as I have lived it until now, not as a woman not as Lesbian, not as a professional. *The High-Tech Protest*

This person combines her personal and professional identity to assess the dangers of the proposed “judicial reform.”

As doctors, we all possess a beautiful soul; I want to believe that. This is something that seems to be part of me. Part of my identity. Therefore, in the end, I found more of a home in the White Coats than in other organizations. *White Coats*

Finally, a respondent tries to analyze the phenomenon and characterize its participants, pointing out the *elite*, without which a modern progressive knowledge-based society could not function.

As a sociologist, I must provide a socio-demographic overview of this influential group. It comprises academics, professionals, high-tech entrepreneurs, lawyers, doctors, psychologists, leading businessmen, and quite a few media people from TV and cinema. It is a good representation of the liberal elite. *Local Centers*

#### 4.1.2 Ability to accept plurality of ideas

The divisions in Israeli society—ethnic, religious, class, level of religiosity, etc. are marked. Still, the activists understand that those divisions, which the government stressed to enable its survival, need to be reconciled, at least to a degree, to enable an Israeli existence.

Controversial issues such as occupation came up in the demonstrations. While many are leaning toward the left, the sentiment of Brothers in Arms is on the other side. *No Academy without Democracy*

It's not that we're pushing some agenda here - we didn't rescue any families from Sderot (Sderot is a right-leaning town, and it was attacked on October 7^th^) because we wanted to ‘convert them' to our political camp.

One idea that attracts me is to do a march that originates in different regions and go up together to Jerusalem for a demonstration of a million! This can be a powerful statement that will demand elections soon. Last month, I met with many rabbis to convince them to support this idea.

You must understand that we are not on the left or right sides. We must live together and, within this framework, love your neighbor like yourself, equality, equal duties, and equal rights - guided by our Declaration of Independence. Because of this activism, we will solve these problems; I am very optimistic. *Brothers in Arms*

A problem we will have to deal with is that there was no representation (in the demonstrations) for all the factions in the Israeli public. We should embrace everyone with open arms. *Black Flags*

An activist in a local center leading a dialogue effort said:

In our conversations with the religious population, I was impressed that the community there is very strong because they meet every Shabbat in a synagogue. Therefore, many of their volunteering activities come from the community and the synagogue. I have a friend here who says: “The demonstrations are the synagogue of the seculars.”

I saw an interesting phenomenon in our activity at Or Akiva (a low-income town), where there was a complete distrust in our intentions; they saw us from day one as demonstrators, not as people who came to talk. In Or Akiva, they perceived the demonstrations as being against; as soon as you demonstrate against the government while I voted for it, it is perceived as if you are against me. This means total identification with the government. And so, there was a whole process, which is still present, of building trust and legitimacy with the ability to be there, to be friends. Many people talk about unity, but they think about unity of opinion, unity with those who agree with me - not unity for a common goal despite our diversity.

The complementary thing that jumps out is the issue of social identity. We are in an age where we look at who is saying something before listening to what you say. The more limited our social identity is, namely, we only belong to one group, the stronger the bond with the group grows because otherwise, we will be lost. Someone said, “If you have nowhere to go back to, then you really have nowhere to go.” *Local Centers*

### 4.2 Belief systems underlying their activity

#### 4.2.1 Guiding values and ideologies: democracy, equality, human rights

When discussing values, our interviewees realize that the proposed “judicial reform” is the tip of the iceberg. Those promoting it have a broad agenda, which needs to be countered by protesting, as well as by fixing an incomplete democratic state infrastructure (i.e., Israel does not have a constitution), which needs to be accompanied by an educational effort.

We wanted to build some kind of infrastructure that would guarantee a Jewish, democratic, and liberal Israel for generations… In the future, we want to influence [this reality] through education or preparatory schools.

We said that even if we succeed in disrupting the ‘reform,'…We will not go out of business. We saw how fragile [our democracy] is, and how much we need to strengthen and protect it in the form of a constitution, as well as education.

We define ourselves in the long run as a civic organization that wants to partner in changing conditions in Israel in the direction of a liberal democratic state, and this is what we do today. *Brothers in Arms*

The idea that the defense of democracy comes from people who have served in the army and security forces has to do with the fact that the Israeli army is based on reservists, namely citizens who may also hold senior positions in the economy and society. They are concerned about introducing significant structural changes in the regime without having broad public approval and the implications of such measures on society and the army.

I come from an ultra-Orthodox family, so the environment of my values was very religious. While I've moved away from that lifestyle, I still cherish its emphasis on generosity, community, and the Jewish principle of leaving the world a better place, encapsulated in Tikkun Olam (Repairing the World). *The High-Tech Protest*

That comment is indicative of the many faces of Judaism. While the Orthodox and the National Religious movements stress a narrow definition and interpretation of Judaism, focusing on the sacramental commandments and on the notion of settling the land, it is important to remember that Judaism has a universal message. A major voice for widening the scope of Judaism and calling for Jewish religious pluralism is Hartman (2011) who advocates a more inclusive and inviting framework for the modern Israeli engagement of the Jewish tradition—a framework that is neither negating nor ignoring non-Jewish contributions to world thinking, in the spirit of the Middle Ages philosopher Moses Maimonides. It furthermore stresses the social and justice foundations of Judaism, which send a universal message.

We aimed to advocate for democracy in Israel and for the preservation of an education that is liberal, humanistic, egalitarian, and pluralistic and will resist intolerance. Our slogan: “There is no education without democracy,” simplified our message, appealing to any parent seeking such a liberal education for their children. *Local Centers*

#### 4.2.2 The occupation

The issue of the occupation, closely tied to human rights, democracy, equality, etc., is controversial, and not all those who participated in the protest were ready to support a slogan condemning the occupation. Some groups held banners criticizing the occupation during protests, but speakers during demonstrations usually avoided touching on this subject. The controversy regarding occupation has to do with the fact that it is related to two dimensions of the Israeli existence: The first is the religious belief that the entire land of Israel/Palestine is God-given and cannot be negotiated. The second has to with security—the idea that the occupation is needed as long as there is no peace between Israelis and Palestinians—the idea that the territories are a bargaining card for future peace negotiations. While that argument does not necessarily lead to building of Jewish settlements in the occupied territories, the notion, that was developed in the pre-state era, that the Israeli border runs along the line where the civilian population lives, provided a sort of legitimacy for the Israeli governments to allow the building of settlements in the West Bank in the past 50 odd years. The rationale for such a policy was the idea that in future negotiations, land on which settlements are built, could be swapped. The duality of this issue makes it controversial, and the demonstrations leaders tried not to emphasize it before the war.

Many of us have the same opinion against occupation. It is an issue that was often controversial during the protests because of the fear that it would drive away activists and harm the protest. *No Academy without Democracy*

We fear a Halachic state (driven by Jewish religious laws) and the legitimation of the settlements. *The Black Flags*

### 4.3 Leadership

#### 4.3.1 Preparedness to contribute to the public sector and civil society

Judicial reform seems to have touched many people's sensitive nerves. It awakened not only their opposition to that proposed regime change but also their general level and volume of activity in the public sphere. The following quotes indicate the necessity and readiness to invest more in contributing to society rather than focusing on oneself.

Some people didn't contribute anything to society until a year ago and now claim to contribute one day a week, asserting their role as partners. Everyone must find their niche. I will not go into politics; there are those who will.

At the beginning, we had the protest: Stopping the planned coup d'état by the Netanyahu government. We thought, two or three months, and it would be over. No one planned to be a social activist. Suddenly, on October 7^th^, many people realize that there is a long-term mission here. *Brothers in Arms*

I've always wanted to contribute. I was in the Girl Scouts and an officer in the army. I have an urge to go back, leaving the rat race to make money and making sure that, in the end, it will be a good place to live here. So, the sense of mission, the desire to feel that you are doing something good, is very significant for me. Still, I can't tell you if I'll be active in an NGO or perhaps moving to the public sector, possibly becoming an official in the Treasury. My generation realizes the importance of seeking to build Zionism 2.0”. What is it? I don't know yet, but I'm sure great changes will occur here. *Building an Alternative*

I personally had a dilemma a few months ago. It has always been clear to me and everyone around me that I will be a member of the Knesset one day, so, in the context of my activity in the protest, the question was: did the time come? One guy told me: “Yes, you should, once you decide (to run), talk to your wife and hire a public relations person.” *The Black Flags*

I believe that what happened to us now is this realization that if we don't become activists, we simply won't be here. We set an example for our children, who must be activists, too. One more thing: I was in Bnei Akiva (youth movement of the National-Religious movement); I saw Gush Emunim (the settlers' movement) and how they promoted their ideas that they have to reach positions of control everywhere: In the army, the police, the public administration. *The High-Tech Protest*

All this activity happens while everyone involved in the protest must also keep their job and build a career and a family. A huge advantage is the people you're exposed to - the real activists. That in itself should be a reason to keep fighting and not give up. *Local Centers*

If we want it to improve this country, the academy should be more involved, and knowledge-based decisions need to be the norm in state institutions. I hope we will succeed because I have a daughter in the army. She wouldn't have come with me if I had moved to the US now, so I'd better stay here. *No Academy without Democracy*

#### 4.3.2 Patriotism expressed in both protest and support activities

This country is important to us, and we need to mobilize despite the complexities of war and having divergent opinions. However, we agreed that what is happening in the country cannot be ignored, and we need to support the populations that need help. *Brothers in Arms*

My job now (in the civil headquarters, which coordinates support for evacuees) is not to provide civil assistance to needy populations. It is to bring the country back to sanity, and that means replacing the government as much as I can influence that. *No Academy without Democracy*

The idea that the protest and the support are motivated by the same patriotism is intriguing. Still, the finding here is evident. The feeling of the need to protect the country and society from internal and external threats is typical of Israelis.

## 5 Discussion: a new serving elite in the making?

We conducted this study against the dramatic 2023 events in Israel, which saw a massive mobilization of protesters for 9 months in all parts of the country. They protested in defense of democracy in light of plans by the government to change the rules and create a de facto authoritarian regime. Many of the same civil society organizations that mobilized protesters changed their orientation overnight after the Oct. 7th Hamas attack and turned into a system of aid that collected needed items and provided services to a variety of affected populations during the war. The instant transition from protest to service delivery is unique and deserves studying from various angles. In this paper, we chose to study the leadership of the organizations that participated in this dual effort, their identities, values, commitment to society, and their preparedness to invest in it. We use the concept of *Serving Elite* to do so.

As we show, the idea of an elite in a modern society entails, first and foremost, *responsibilities* rather than privileges. In a meritocracy, somebody has to make decisions on behalf of the collective, where the idea is that the best people should be in positions where they can make those decisions. Very often, people who have proven themselves and deserve trust get credit for making such choices based on their deeds for the collective. In certain ideologies, this includes readiness to be put in dangerous positions and risking one's life.

We present the concept of *Serving Elite* within the Israeli context, both from a historical and a contemporary perspective. It started with the Kibbutz movement in the early 20th century, beginning in the Ottoman era, which established Kibbutzim. That movement set an example for Israeli society through its conduct based on equality, democracy, and patriotism. Also expressing its position was its disproportional share of soldiers killed in the Israeli wars. Based on these facts, their representatives filled many Knesset and Cabinet seats. In the 1980s, with the loss of power of the Labor Party and the rise of the right-wing Likud party, and with processes of individualism that replaced the collectivism, which was representative of the Israeli society, the Kibbutz movement's role as a serving elite waned, giving way to the National-Religious movement rooted in Messianic beliefs. Their members prioritize settling throughout the holy Land of Israel/Palestine, particularly in the West Bank, despite the dangers posed by daily interactions with Palestinians and the risks posed to Israel's democracy and international standing. Similarly to the Kibbutz movement, they have a disproportional share of soldiers killed in Israeli wars as well as a disproportional number of members in the Knesset, the cabinet and public administration.

While the protests of 2023 focused on the “judicial reform,” the government that intended to pass it was maneuvered by the National-Religious political forces. Thus, the protest was not only against the “reform” but also against the danger of a national-religious ideology taking over and controlling society. Thus, the main argument of the paper is that the protest of 2023 has created a foundation for a new serving elite—a Liberal one composed of professionals, academics, high-tech entrepreneurs (leading the Israeli economy), former security forces personnel, etc.—the foundation for a modern liberal society and economy. It can be seen as a *counter-elite* to the National-Religious one, as it deals first and foremost with values that govern society. Their activities during the first part of the year showed their devotion to preserving Israel's democratic character. Despite being labeled “anarchists” and “traitors” by the government and its supporters, their actions since October 7th, when they transitioned from protesting to supporting populations affected by war, have demonstrated their sense of responsibility and patriotism. This would-be Liberal serving elite, a coalition formed during a crisis, is characterized by their commitment to ideals that are under threat. Our study only begins to outline their identities, backgrounds, values, and dedication to the public. Unlike past elites focused on settling the land, this liberal elite prioritizes education and preserving liberal-democratic values. Still, these values were added to a loyalty and an uncompromising commitment to the Israeli state. This was stressed by the fact that most demonstrators came with Israeli flags in their hands and each demonstration started with the singing of the national anthem. It was also manifested by their response regarding activism and the need to contribute to society, if it is to remain democratic and liberal. Given that such response comes from individuals who were recently focused primarily on their careers and families, provides an indication regarding a change of personal priorities as well as a sense of social and national responsibility. As the clash between religious-Messianic and democratic-liberal ideologies shifts to the political arena, the confrontation between these two serving elites will shape the future of Israeli society.

In addition to this case's contribution to understanding Israeli society during a major crisis, the study contributes conceptually to the literature on elites, specifically *Serving Elites*. Based on our findings, we can portray the following attributes of a serving elite and offer a working definition of this concept:

1. *Shared Ideology* is the central organizing and mobilizing principle that also allows for bridging differences in views and identities between groups included in the elite.
2. *Identity*—Self-perception as an elite entails awareness of the identities and their related traits that put them in a preferable position to influence society and polity.
3. *Responsibility*—A sense of individual and collective responsibility for the wellbeing of the entire society; a willingness to undertake roles and positions in public and civil service, including in the military.
4. *Activism*—A willingness to translate ideology and responsibility into action on behalf of the collective. Taking personal, financial, and even physical risks for this activism and serving as role models for peers and the next generation.

These components are clearly compatible with the emergent serving elite that we analyzed here and the two historic serving elites that we discussed throughout the article—The Kibbutz and the National-Religious movements. We believe that with further research, we will find that this conceptualization and its components are relevant beyond the Israeli case and can serve as a valuable concept for analyzing social and political change, elites, and other related phenomena. The “serving elite” concept, not frequently discussed in the literature on elites, can elucidate significant societal dynamics with far-reaching implications. A comparison of its specific attributes in other contexts would strengthen its validity.

## Data availability statement

The raw data supporting the conclusions of this article will be made available by the authors, without undue reservation.

## Ethics statement

The studies involving humans were approved by Dr. Gila Oren and Dr. Nili Karako-Eyal. The studies were conducted in accordance with the local legislation and institutional requirements. The participants provided their written informed consent to participate in this study.

## Author contributions

BG: Conceptualization, Data curation, Formal analysis, Investigation, Methodology, Project administration, Writing – original draft. HK: Conceptualization, Data curation, Investigation, Methodology, Writing – review & editing.

## Footnotes

^1^A liberal arts college aiming to develop future functionaries in Israel's civil service.

^2^An instant messaging application for smartphones.

^3^In order to protect their identities, we promised our interviewees not to disclose their names.
