# Supplementary material for: Israel's civil society 2023 from protest to aid provision – a serving elite perspective
Source: Front Sociol. 2024 Jul 9;9:1417687. doi: 10.3389/fsoc.2024.1417687 (PMC11265563; doi:10.3389/fsoc.2024.1417687)
Supplement: Supplementary file 1 [file Data_Sheet_1.docx]

# Appendix. Detailed Description of Organizations Interviewed and their Activities

*Brothers and Sisters in Arms (Achim La’Neshek)* is an organization of military reserve men and women from various units in the Israel Defense Forces (IDF), which operated as part of the protests against the Judicial Reform. Since October 7th, 2023, the organization has suspended all political and protest activities and devoted itself full-time to aid and relief under  *Brothers and Sisters for Israel***.** The organization aimed to stop the government’s legislative measures and strengthen Israeli democracy. During the protest phase, the organization operated in a variety of ways, including demonstrations in front of the homes of ministers and members of the Knesset, “vigils” that included discussion positions, advocacy in Israel and abroad, and disruption and protest actions, such as hanging giant signs on cliffs and bridges. They also initiated the 3-day march from Tel-Aviv to Jerusalem with tens of thousands of participants, ending it in a big demonstration near the Knesset and blocking the Haifa port (with small boats). Another central activity was signing a statement that they would stop volunteering for reserve duty until the planned legislative measures were stopped (which did not mean refusal to serve when called in an emergency). They funded the organization’s operations through donations from the public and the sale of its branded equipment (shirts and hats).

After October 7^th,^ they formally incorporated to deal with donations for the victims of the war. On the morning of October 7^th^, they made an official announcement, declaring an immediate halt to all protest activities. They called reserve soldiers to report to their units to defend the country. On the same day, the infrastructure previously used for the protest was converted to assist security forces and provide aid to survivors of the Hamas massacre. The organization established dedicated logistic centers in Tel Aviv and Beit Kama (not far from the towns and Kibbutzim around Gaza), where they coordinated numerous initiatives with the help of tens of thousands of volunteers nationwide to collect needed items and evacuate thousands of families from the conflict zones to safe areas. Additionally, they made efforts to secure housing solutions for displaced families and to assist the security forces in their quest to locate missing individuals, employing advanced technological resources for this purpose. They organized assistance to the families of murdered, missing, or kidnapped individuals, farmers near war zones, and small businesses affected by the war. Moreover, they set up a voluntary transportation system to facilitate the movement of reservist soldiers and their families. They built around 200 kindergartens for internally displaced persons.

*Women Building an Alternative (Bonot Alternativa)* is a feminist activist organization that seeks to promote gender equality, empower women, and raise awareness of violence against women. The group started in 2020; it brought together businesswomen, entrepreneurs, female CEOs, media women, social activists, and heads of women’s organizations. The organization engaged in protests around feminist issues before 2023. They understood that judicial reform would create a detrimental situation for women, and the organization decided to join the demonstrations. Within the demonstrations, they focused on the issue of equality for women. It was essential for them that women would be among the speakers, stressing their professional backgrounds. As part of the protest against the judicial reform, the movement initiated the “Handmaids’ Protest” inspired by the TV series based on Canadian author Margaret Atwood’s book, *The Handmaid’s Tale*. Hundreds of women dressed in red robes solemnly marched in silence, symbolically representing slave girls, in many locations during the protests, and it became a trademark for the organization. It was a very successful tactic that drew much attention. The organization grew to roughly 100,000 members; based on their activity in Bonot, some members decided to run in the municipal elections. Their focus is to expose discrimination against women where it occurs and create public awareness of it. Activities included vigils in front of rabbinical courts (where women have an inferior status) and protests against the new government dismissing women directors-general in government ministries. They separate themselves from organizations that provide specific services to women, e.g., rape crisis centers, focusing on the political aspects of equal representation. They raised (before October 7^th^) some 10 million Shekels.
Since October 7^th^, the organization swiftly repurposed its entire infrastructure for relief efforts. To this end, they collaborated with Brothers and Sisters in Arms and other organizations that previously focused on protest activities. They were involved in sorting through tons of donations of food, clothing, and hygiene products, packing boxes for delivery to the injured, soldiers, and families who lost their belongings in the onslaught. As a women’s protest organization, they plan to continue fighting for women’s rights after the war. They were involved in exposing the sexual abuse by the Hamas terrorists on the international scene, had news conferences, and went to the White House.

*No Academy without Democracy*. Leaders of various scientific communities led by faculty members of universities and colleges have participated in demonstrations against the judicial overhaul. Their presidents have threatened to resign in protest. Israeli universities have encouraged staff and students to participate in demonstrations against the legislation. In August 2023, the heads of public research universities and members of the National Council for Research and Development warned of “destructive” consequences for the “future of scientific research” in the country due to the government’s judicial overhaul. In a letter addressed to the Prime Minister and Education and Science Ministers, the academics cited growing signs in the past weeks that testify to destructive developments that could harm the scientific resilience of Israel. They wrote and disseminated position papers and reactions to events and proposed policies. They organized a significant march in Jerusalem and planned not to open the academic year. When the Minister of Justice planned to visit the Tel Aviv University campus, they planned to disrupt his visit. They also blocked a senior political appointment with the Council on Higher Education.

After October 7^th^, the group was active in the international scene, trying to counter antisemitic and pro-Hamas trends in universities abroad. Members joined Brothers and Sisters in Arms in their diverse activities. Some of their members actively identified missing persons from the Nova event using diverse sources and hi-tech technologies, going over thousands of pictures and movies.

*No Education without Democracy* is an organization of educators, parents, and students who regularly demonstrated outside educational institutes in dozens of locations against the government’s planned overhaul of the judicial system, which would severely threaten democracy and leave individual rights unprotected and minorities undefended. They held demonstrations at schools and kindergartens under the banner “There is no education without democracy.” They were concerned that plans to change school curricula would leave them hostile to aspects of existing content teaching pluralism and tolerance.

*White Coats****.*** An organization of doctors, nurses, and other health professionals led by the chair of the doctors’ union. They protested against the danger posed by the proposed judicial reform to public health and universal accessibility to health services. They started as professionals providing an emergency health focus in the demonstrations, providing first aid in case of injuries. They also offered mental health assistance to protesters. They conducted activities (mostly rallies) in hospitals and on the national level and held a rally at Jerusalem’s International Convention Center, with over 3,000 participants.
Soon after the break of the war on October 7^th^, they noticed an urgent need for medical equipment. Members of the organization used their connections to purchase medical equipment in different countries, coordinating the logistics with Brothers and Sisters in Arms. They raised funds from private donors to purchase and transport the equipment via private planes to Israel. They provided hundreds of packages worth over $3 million. They were also concerned with the kidnapped persons and were in contact with the Red Cross to provide medication to the kidnapped.

*High Tech Protest.* In addition to joining the general protests against the proposed judicial reform, members of the hi-tech industry joined other protest leaders in announcing an “Economic Resistance Day.” Their focus was the damage that the new legislation would cause to the Hi-Tech industry by discouraging foreign investment and deteriorating Israel’s human capital. After October 7^th^, hundreds of volunteers with tech experience were using advanced tools to verify the identities of those suspected abducted. They used their networks to collect items and provide services to needy populations.

*Local Centers* (we interviewed activists in two such centers)*.* Regional and local nuclei of the protest were established in over 60 locations outside the main cities, which organized synchronous protests with the same content. Often, speakers rotated from location to location to expose the demonstrators to different voices during the protest. In addition to protests, these regional nuclei also engaged in dialogs with people in communities where there was support for the reform. Activities included opening a table in a central square, offering a flower to passers-by, and engaging them in a conversation about life and democracy. Some local centers were organized around a school community of parents, children, and school staff, focusing on the threats to liberal-democratic educational content in the public education system posed by the judicial reform. They were involved in various activities, such as distributing written materials and T-shirts with pro-democratic messages and demonstrations in front of ministers’ homes and in major intersections. Since October 7^th,^ individuals from the local centers volunteered in other organizations. The groups that engaged in a dialog with the population continued to do so.

*Black Flags*/Kaplan Force. This organization started in 2020 as a protest against PM Netanyahu, focusing on his corruption. They hung banners on bridges and intersections and demonstrated in public places. They had some 80,000 members in WhatsApp groups. They were perceived as an extreme element, far-left anti-occupation. In 2023, they joined the nationwide protest movement and renamed themselves Kaplan Force (based on the name of the street in Tel Aviv where the protests took place). Prof. Shikma Bressler, one of its leaders, became a major spokesperson and “face” of the entire protest movement. On October 7^th^, they joined the efforts to collect needed items and funds for the soldiers and evacuees using their networks.

*Crime Minister***.** The movement started in 2018. Movement members protested Prime Minister Netanyahu every Saturday night, both outside his residence in Jerusalem and in Tel Aviv. The protest focused on the fact that the prime minister is on trial and accused his government of corruption. They joined the protests in 2023 but accused the protesters of being too soft as they did not demand the resignation of the Netanyahu government. They advocated much more radical and confrontational measures, such as blocking Ben-Gurion airport. After October 7^th,^ they did not join the support and relief efforts and criticized the Black Flags organization that Brothers in Arms swayed to follow a less radical line of protest.
